# Assessing Uncertainty in High-Resolution Spatial Climate Data across the US Northeast

**DOI:** 10.1371/journal.pone.0070260

**Published:** 2013-08-01

**Authors:** Daniel A. Bishop, Colin M. Beier

**Affiliations:** 1 Department of Forest and Natural Resources Management, College of Environmental Science and Forestry, State University of New York, Syracuse, New York, United States of America; 2 Adirondack Ecological Center, College of Environmental Science and Forestry, State University of New York, Newcomb, New York, United States of America; University of Oxford, United Kingdom

## Abstract

Local and regional-scale knowledge of climate change is needed to model ecosystem responses, assess vulnerabilities and devise effective adaptation strategies. High-resolution gridded historical climate (GHC) products address this need, but come with multiple sources of uncertainty that are typically not well understood by data users. To better understand this uncertainty in a region with a complex climatology, we conducted a ground-truthing analysis of two 4 km GHC temperature products (PRISM and NRCC) for the US Northeast using 51 Cooperative Network (COOP) weather stations utilized by both GHC products. We estimated GHC prediction error for monthly temperature means and trends (1980–2009) across the US Northeast and evaluated any landscape effects (e.g., elevation, distance from coast) on those prediction errors. Results indicated that station-based prediction errors for the two GHC products were similar in magnitude, but on average, the NRCC product predicted cooler than observed temperature means and trends, while PRISM was cooler for means and warmer for trends. We found no evidence for systematic sources of uncertainty across the US Northeast, although errors were largest at high elevations. Errors in the coarse-scale (4 km) digital elevation models used by each product were correlated with temperature prediction errors, more so for NRCC than PRISM. In summary, uncertainty in spatial climate data has many sources and we recommend that data users develop an understanding of uncertainty at the appropriate scales for their purposes. To this end, we demonstrate a simple method for utilizing weather stations to assess local GHC uncertainty and inform decisions among alternative GHC products.

## Introduction

A growing demand for high-resolution spatial climate information by researchers, educators and decision-makers has led to the creation of various gridded historical climate (GHC) data products (e.g., [1]–[9]). These GHC products are often freely available and easily incorporated in geographic information systems (GIS) and are now being widely used for mapping and estimating climate conditions at local and regional scales. Applications of these data include regional bioclimatic modeling [10], ecosystem simulations [11], and the statistical downscaling of global climate model (AOGCM) forecasts [12], [13].

Despite rapid growth in use of high-resolution GHC products in research and decision-making, there remain several issues that have received little attention. First, GHC users without formal training in climatology or geospatial modeling may be unaware of the inherent uncertainty associated with these products [14]. Although GHC data are generated using robust techniques to assess uncertainty, few (if any) GHC products are provided with their corresponding error or uncertainty estimates at grid resolution [2], [8], [15]. Instead of treating the data as model outputs with associated uncertainty, it appears many GHC users treat them as ‘true’ climate information – i.e., an accurately measured independent variable – in statistical, spatial and simulation models. Yet these GHC products are model outputs that typically have increasing uncertainty at higher resolutions, creating a ‘resolution vs. realism tradeoff’, in which finer grain maps appear more intuitively accurate but cannot be validated [14]. Model errors are not uniform across space or time, but tend to be spatially and temporally complex, representing error propagation from sources related to both measurement and modeling [14], [16], [17].

One of these complexities results from the strong influence of elevation on climate itself. As a result, the modeling techniques that are used to represent continuous climatic variation across complex terrains tend to be heavily dependent upon elevation as a predictor variable [1], [3], [5], [7], [8]. Spatial interpolation of daily temperature is strongly influenced by elevation, more so for maximum temperatures than minimum temperatures [18], because the former are particularly sensitive to the effects of temperature inversions in inland areas [3].

Another issue facing data users is that many different GHC products exist and there are few, if any, clear guidelines for choosing among them. At face value, GHC products generated at similar resolutions may appear quite similar when mapped, but have significant disparities in their predictions [17]. In general, a robust approach would be to compare multiple GHC products using their relative uncertainties at locations of interest (model comparison) and to determine their level of agreement with each other and with reference data. Few studies of this type have been done (but see [14], [19]) and to our knowledge a regional-scale validation and comparison of two or more GHC products has not been done. The PRISM (Parameter Regression on Independent Slopes Model, PRISM Group, Oregon State University, Corvallis, OR; www.prism.oregonstate.edu) product has been subject to cross-validation analyses, measuring the difference in station observations and GHC estimates after the station has been removed from the model [3], [4]; but, this method tends to favor models that excessively smooth the results, ignoring local climatology. A basic ground-truthing analysis, which can be performed by data users, may be especially useful when multiple GHC products disagree in data-sparse areas, or seem very different from local observations, and can inform specific choices on GHC usage in research and decision-making.

In this study we compared the predicted means and trends of two 4 km GHC temperature products – PRISM [2] and NRCC (Northeast Regional Climate Center, Cornell University, Ithaca, NY; www.nrcc.cornell.edu) [8] – for the same region (US Northeast) and time period (1980–2009) with the observed temperature means and trends from a group of weather stations that were incorporated in both products. Given the reference data was directly used in the GHC models, we could have expected to find negligible differences between station observations and model (map) predictions. On the other hand, previous analyses of PRISM and NRCC indicated there might be systematic sources of uncertainty in the US Northeast: cells located at higher elevations and along coastlines appeared to have consistently larger cross-dataset bias, and a ground-truthing at a very high-elevation station suggested very large prediction errors [17].

To better understand this situation as GHC data users, our objective was to gain some insight on sources of uncertainty and make further comparisons between the PRISM and NRCC products. We evaluated effects of landscape factors such as elevation and distance to coast on prediction errors using information-theoretic modeling [20]. Other error sources, such as the coarse-scale (4 km) elevation models used by GHC models, were also assessed. Lastly, we demonstrate a method for using local weather stations to interpret areas of significant disparity between GHC predictions [17] and to inform decisions among alternative GHC products.

## Methods

### 1. Gridded Climate Data Products

Characteristics of the PRISM and NRCC gridded data products are summarized in Table 1; each is briefly described here. The PRISM product is generated using moving-window, elevation-dependent regression models that interpolate a large group of high-quality instrumental records to a 4 km grid across the US states from 1895-present. PRISM incorporates a complex weather station weighting system including spatial topographic facets, coastal proximity, topographic position, effective terrain height, elevation, station distance, and station clustering [2], [4]. The PRISM temperature and precipitation climate products are freely available GHC products that serve as the official climatological data of several US agencies.

**Table 1:** NRCC (Northeast Regional Climate Center, Cornell University, Ithaca, NY; www.nrcc.cornell.edu) and PRISM (Parameter Regression on Independent Slopes Model) product summary [17].

Data Product	Resolution		Domain		Data Sources		Methods
	Temporal	Spatial	Temporal	Spatial	Primary	Secondary	
PRISM [2]	Monthly	4 km	1895-present	Contiguous 48 US states	Station records	4 km elevation model;geographic facets^1^	Regression models based on elevation, facets, using an expert system with weighting schemes
NRCC [8]	Daily	2.5 arc-min (4 kmat 45°N)	1979-present	US Northeast (NY, VT, NH,ME, MA, RI, CT, PA, NJ)	North American RegionalReanalysis^2^; Selected COOP^3^Stations	2.5 arc min (4 km)elevation model	Downscaling of NARR to 4 km elevation model; Bias correction within a defined radius using selected COOP stations

1 Geographical facets in PRISM represent combinations of slope, aspect and elevation generated by the PRISM system for regression modeling and interpolation.

2 The North American Regional Reanalysis (NARR) is a 50 km resolution model output generated by a regional meteorological model that has been back-calibrated with observed satellite, radar and ground-based instrumental records [21].

3 NOAA Cooperative Observer Network (COOP) weather stations.

**Figure 1:**
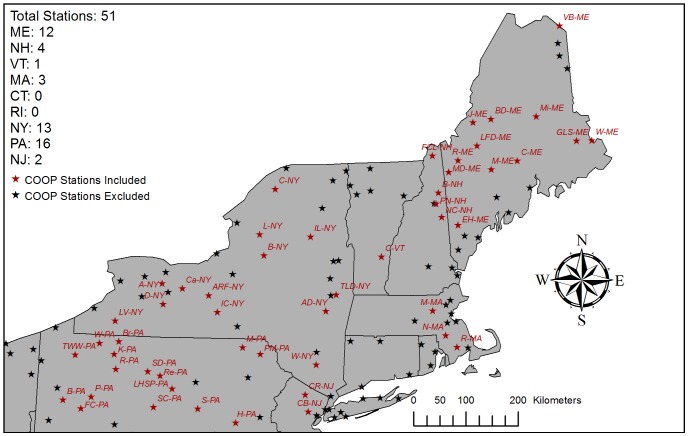
Map of study area and COOP stations. Station density for each state listed in upper left-hand corner: Maine (ME), New Hampshire (NH), Vermont (VT), Massachusetts (MA), Connecticut (CT), Rhode Island (RI), New York (NY) and northern portions of Pennsylvania (PA) and New Jersey (NJ). Stations are assigned unique identifications listed in Table S1.

The NRCC product is generated by downscaling and bias-correcting the 40 km resolution outputs of the North American Regional Reanalysis (NARR). The NARR is based on a regional meteorological model that has been back-calibrated using satellite observational data, radar, and weather stations [8], [21]. NRCC downscales the NARR using a digital elevation model (DEM) at 2.5×2.5 arc-minute resolution, which is approximately 4 km at 45°N. Bias correction in the NRCC is based on a subset of NOAA Cooperative Observer Network (COOP) stations in the region (Fig. 1).

The two 4 km GHC products have nearly identical raster geometry (within 0.00001 degrees congruence) across the US Northeast region, which is defined by the extent of the NRCC spatial domain, containing all of Maine (ME), New Hampshire (NH), Vermont (VT), Massachusetts (MA), Connecticut (CT), Rhode Island (RI), New York (NY) and portions of northern Pennsylvania (PA) and New Jersey (NJ). Each product uses a different DEM, employing different methods to smooth to a similar resolution ∼4 km (Table 2). DEMs present an elevation estimate for each gridded cell. As DEMs represent a continuous area rather than a discrete point, these elevation estimates may differ from a point’s exact elevation depending on surface roughness and slope.

**Table 2:** PRISM and NRCC digital elevation model (DEM) summary.

Data Product	Spatial Resolution	Source	Methods
PRISM	2.5 arc-minute	EROS Data Center’s 3-arc-secondDEM	Mosaiced & resampled to 15-arc-second resolution; Smoothed with modified Barnes filter [34]
NRCC	2.5 arc-minute	NOAA NGDC 30-arc-second(∼1 km) DEM	Mosaiced & resampled to 150-arc-second resolution

All computations including statistical models were conducted using the R Project [22]. The R packages ncdf [23], raster [24], lme4 [25], gdata [26], sp [27], and spatstat [28] were used.

### 2. Station Validation Analysis

Temperature means and trends from the PRISM and NRCC products were compared with concurrent observations (1980–2009) from a subset of Cooperative Observer Network (COOP) stations across the US Northeast. Of the 116 COOP stations in the region, we analyzed prediction error at the 51 stations that were 1) used, to our knowledge, by both PRISM and NRCC, and 2) did not contain any missing or erroneous monthly data (Fig. 1). Twenty of the COOP stations we utilized are part of the US Historical Climatology Network (US HCN; [29]). In this paper, individual stations are referenced by their unique identifiers (Table S1). The remaining 65 stations not included here were not used in NRCC because of measurement schedules [8], but may have *potentially* been used in PRISM. It is important to note that we say “potentially been used” because PRISM draws its data from numerous stations but not all model runs utilize all stations with equal weighting.

Trend magnitude estimates for PRISM, NRCC and weather station records were calculated using the least squares regression slope coefficient to estimate rate of change. The slope coefficients were multiplied by 10 to present trend magnitudes as decadal rates (°C decade^−1^).

Prediction errors as simple differences (predicted–observed) were calculated at the 51 COOP station points using the 4 km raster cell containing the coordinates of each station. The monthly-resolution prediction errors were averaged annually for each station, producing 8 sets of prediction errors at each weather station: 2 GHC products (PRISM, NRCC) ×2 temperature variables (TMin, TMax) ×2 statistics (mean, trend magnitude). The monthly-resolution prediction errors were also averaged seasonally into winter (DJF), spring (MAM), summer (JJA) and fall (SON). Probability distributions of the errors were compiled for each set of seasonal and annual outputs, from which moments were estimated (mean, median, standard deviation, etc). A pooled probability distribution combining PRISM and NRCC errors for a given variable (e.g., mean TMax, trend TMin) was also compiled for outlier analysis.

### 3. Sources of GHC error

We used information theoretic modeling [20] to evaluate effects of landscape factors on error at the station locations. The factors evaluated were latitude, longitude, elevation and distance to coast. Elevation, latitude and longitude were derived from the station’s NCDC metadata. Distance to coast was calculated in ArcGIS for each station using a coastline shapefile provided by the NOAA NGDC (www.ngdc.noaa.gov/mgg/coast).

To reduce collinearity, a correlation screening was conducted using the Pearson product-moment test. Based on these results, longitude was excluded from model selection because it was highly correlated, using the Pearson correlation coefficient (r), with both distance to coast (r = 0.83) and latitude (r = 0.80). The remaining predictor variables (latitude, elevation, and distance to coast) were incorporated in a model selection procedure that evaluated all possible combinations of variables, including null models and interaction terms (Table 3). Response (dependent) variables were the prediction error sets for seasonal temperature means and trend magnitudes based on differences with station observations (n = 51) averaged over 1980–2009. For this analysis, model selections were run based on the 16 error sets summarized for each GHC product: 4 seasons×2 variables (TMin, TMax) ×2 statistics (mean, trend magnitude).

**Table 3:** Temperature prediction error models.

Model
A) Null
B) Lat
C) Elev
D) Coast
E) Lat+Elev+Coast
F) Lat+Elev
G) Elev+Coast
H) Lat+Coast
I) Elev*Lat
J) Coast*Elev
K) Coast+Elev*Lat
L) Lat+Coast*Elev
M) Elev*Lat+Coast*Elev

Explanatory variables included latitude (Lat), elevation (Elev), and coastal distance (Coast).

General multiple linear regression models were created using all possible combinations of explanatory variables, including a null model and models with interaction terms, and compared using the corrected Akaike Information Criterion [20] adjusted for small sample sizes (AIC_c_). Model explanatory power was estimated using the maximum likelihood pseudo-coefficient of determination (R^2^).

We also evaluated a known source of error in GHC predictions – the use of coarse-scale digital elevation models that poorly represent local topography, especially in complex terrains [7], [8]. Differences between each product’s DEM elevation estimates and actual station elevation were calculated and relationships between GHC prediction error and DEM elevation errors were evaluated using Pearson correlation tests, calculating the Pearson correlation coefficient (r) and the significance level using the test’s p-value (p).

### 4. Evaluating GHC Products at Local Scale

A previous analysis of PRISM and NRCC indicated consistent areas of map disagreement between temperature predictions, for both means and trends, across the US Northeast [17]. To better evaluate these areas of disparity, we developed a three-step method using weather stations as an unbiased reference.

First, we identified areas of spatially continuous and empirically large cross-GHC bias, based on simple differences (PRISM minus NRCC). These cross-dataset bias maps were used to represent areas of PRISM>NRCC (high values; mapped in red) and PRISM<NRCC (low values; mapped in blue). These maps allowed us to examine bias patches, or continuous zones of disagreement between the two products, across the region for temperature means and trends.

Next, we identified stations where one or both GHC product had significant prediction errors. Two types of errors were flagged: ‘large’ were those falling outside ±1 SD of the mean pooled error, and ‘outlier’ were those outside ±2 SD of the mean pooled error. Four sets of large and outlier errors were flagged for mean TMax, mean TMin, TMax trend, and TMin trend based on the corresponding pooled error distributions of PRISM and NRCC for each variable.

Lastly, to interpret which local-scale GHC predictions may be more reliable, we overlaid the bias maps with station locations where large and outlier prediction errors were found. For instance, if the PRISM-NRCC bias map indicated that PRISM was consistently warmer than NRCC (i.e., a PRISM>NRCC ‘red patch’), and there was a large positive prediction error for PRISM at weather station(s) in that area, we can infer that PRISM was too warm. Alternately, if NRCC was shown to have a large negative prediction error at weather station(s) within the PRISM>NRCC patch, NRCC might be inferred as too cold. For each map, up to five stations with the largest absolute bias (PRISM – NRCC difference) were included for reference; a sixth station location where both GHC predictions were similar but erroneous – i.e., large error, low bias – was included to illustrate where neither prediction may be reliable.

## Results

### 1. Prediction Error – Mean Temperatures

Both the PRISM and NRCC 4 km products predicted lower than observed mean temperatures across the US Northeast during 1980–2009, based on seasonal and annual averages of errors calculated on a monthly basis at 51 COOP weather stations (Table 4). The exception was PRISM mean summer TMin error, which was positive but near zero (+0.056°C). Median prediction errors are typically closer to zero than mean prediction errors, indicating that large outliers may bias the mean error estimates.

**Table 4:** PRISM and NRCC temperature mean and trend magnitude prediction error averages (standard deviations) and medians.

		Averages				Medians			
		Means (°C )		Trends (°C decade^−1^ )		Means (°C )		Trends (°C decade^−1^ )	
		*TMax*	*TMin*	*TMax*	*TMin*	*TMax*	*TMin*	*TMax*	*TMin*
	*Annual*	−0.230 (0.37)	−0.145 (0.52)	0.012 (0.18)	0.014 (0.23)	−0.188	−0.125	0.001	0.030
	*Winter*	−0.229 (0.37)	−0.351 (0.67)	0.006 (0.17)	−0.047 (0.32)	−0.195	−0.379	−0.014	−0.057
PRISM	*Spring*	−0.223 (0.39)	−0.143 (0.49)	0.015 (0.20)	0.042 (0.25)	−0.190	−0.069	0.004	0.018
	*Summer*	−0.252 (0.42)	0.056 (0.57)	0.027 (0.22)	0.070 (0.22)	−0.179	0.083	0.013	0.051
	*Fall*	−0.213 (0.37)	−0.143 (0.52)	0.006 (0.18)	−0.008 (0.25)	−0.217	−0.139	−0.026	−0.004
		*TMax*	*TMin*	*TMax*	*TMin*	*TMax*	*TMin*	*TMax*	*TMin*
	*Annual*	−0.197 (0.58)	−0.463 (0.62)	−0.044 (0.11)	−0.025 (0.19)	−0.153	−0.382	−0.010	0.006
	*Winter*	−0.347 (0.47)	−0.775 (0.72)	−0.058 (0.12)	−0.019 (0.25)	−0.304	−0.701	−0.026	0.018
NRCC	*Spring*	−0.161 (0.63)	−0.387 (0.63)	−0.039 (0.13)	−0.020 (0.20)	−0.163	−0.300	−0.015	−0.018
	*Summer*	−0.113 (0.73)	−0.164 (0.68)	−0.037 (0.14)	−0.016 (0.17)	−0.094	−0.122	0.001	−0.010
	*Fall*	−0.167 (0.58)	−0.525 (0.63)	−0.041 (0.12)	−0.045 (0.19)	−0.102	−0.413	−0.011	−0.014

**Figure 2:**
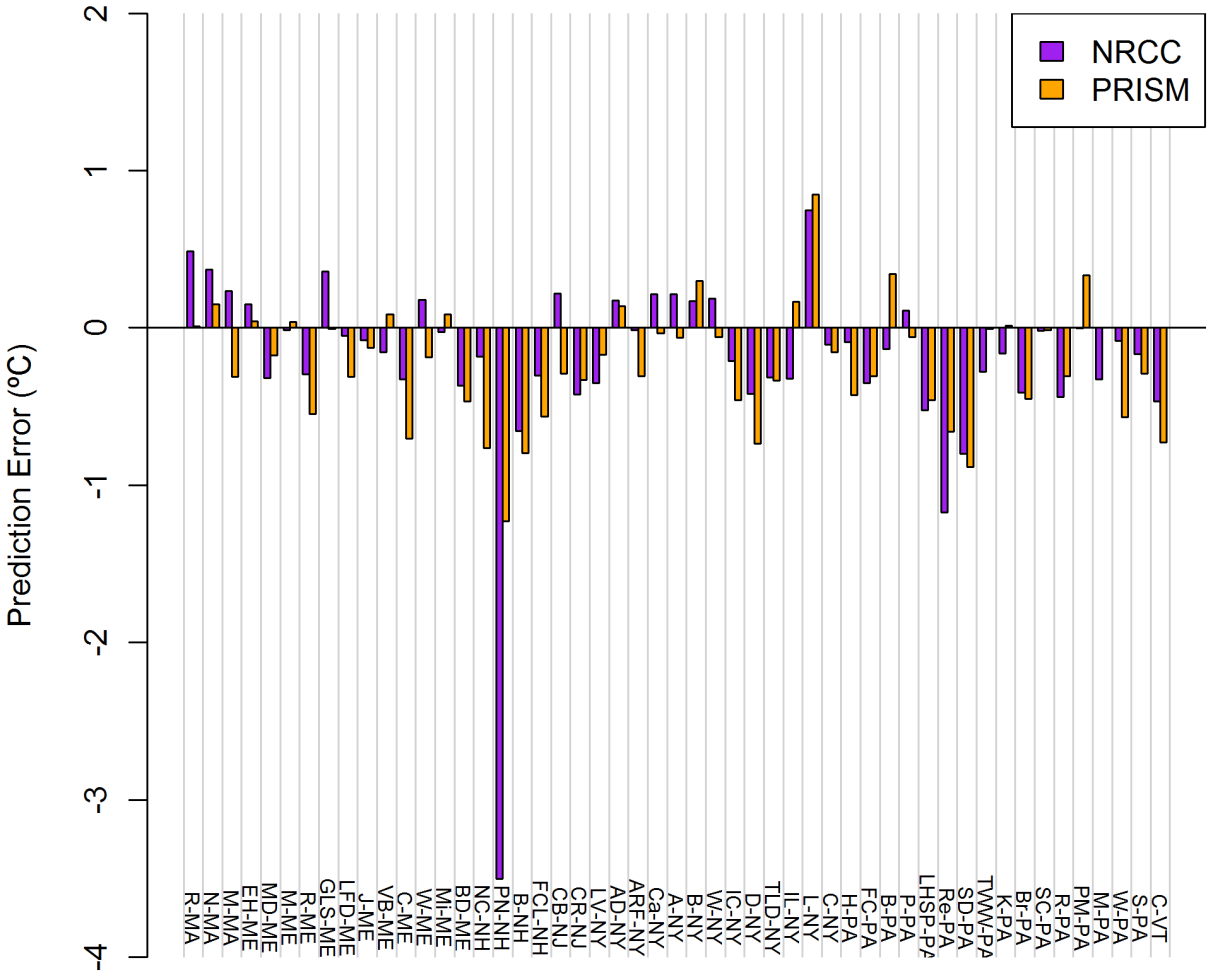
Mean monthly TMax prediction errors for NRCC and PRISM at 51 COOP weather stations. Stations across the US Northeast during 1980–2009. Data presented are averages of 12 monthly errors.

**Figure 3:**
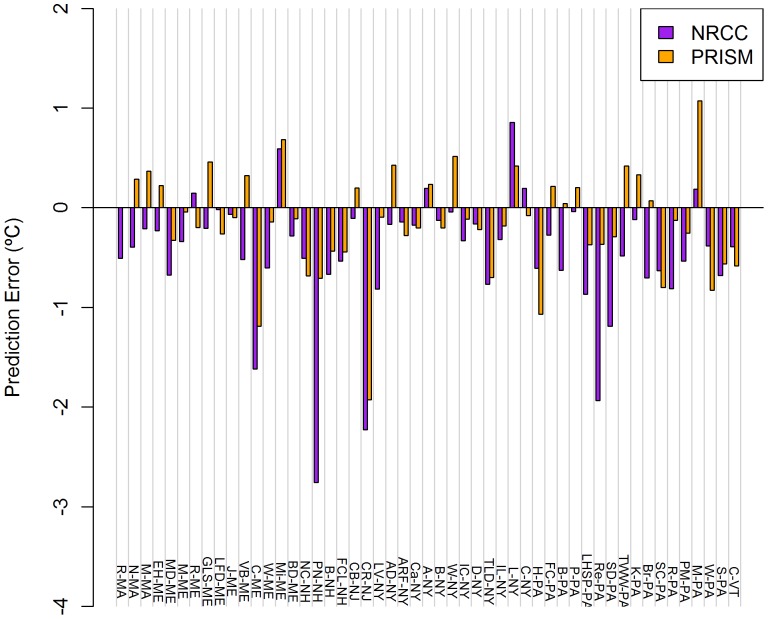
Mean monthly TMin prediction errors for NRCC and PRISM at 51 COOP weather stations. Stations across the US Northeast during 1980–2009. Data presented are averages of 12 monthly errors.

NRCC errors were consistently larger for TMin relative to TMax (Table 4), ranging from −3.501°C (PN-NH) to +0.746°C (L-NY) for mean TMax (Fig. 2) and from −2.756°C (PN-NH) to +0.857°C (L-NY) for mean TMin (Fig. 3). Averaged annually, NRCC predicted lower temperatures than observed at 37 of 51 stations for mean TMax and 45 of 51 stations for mean TMin.

PRISM errors were consistently larger for TMax relative to TMin, with the exception of winter (Table 4). Errors ranged from −1.227°C (PN-NH) to +0.848°C (L-NY) for mean TMax (Fig. 2) and from −1.924°C (CR-NJ) to +1.073°C (M-PA) for mean TMin (Fig. 3). Averaged annually, PRISM predicted lower mean temperatures at 38 of 51 stations for mean TMax and 33 of 51 stations for mean TMin.

### 2. Prediction Error – Temperature Trends

The NRCC product consistently predicted lower magnitude (cooler) trends than observed across the US Northeast (1980–2009) and the PRISM product mostly predicted higher magnitude (warmer) trends (Table 4). For both products, the average trend prediction errors were near zero (<0.050°C decade^−1^), but this was due to much larger positive and negative errors being offset. For example, PRISM had absolute prediction errors ≤0.250°C decade^−1^ at 10 of 51 stations for TMax trends, and 3 of these 10 errors were negative (i.e., a more positive trend was observed than predicted). Both products had their largest prediction error at the same station (M-PA) for TMin trends. Median prediction errors are typically closer to zero than mean errors.

**Figure 4:**
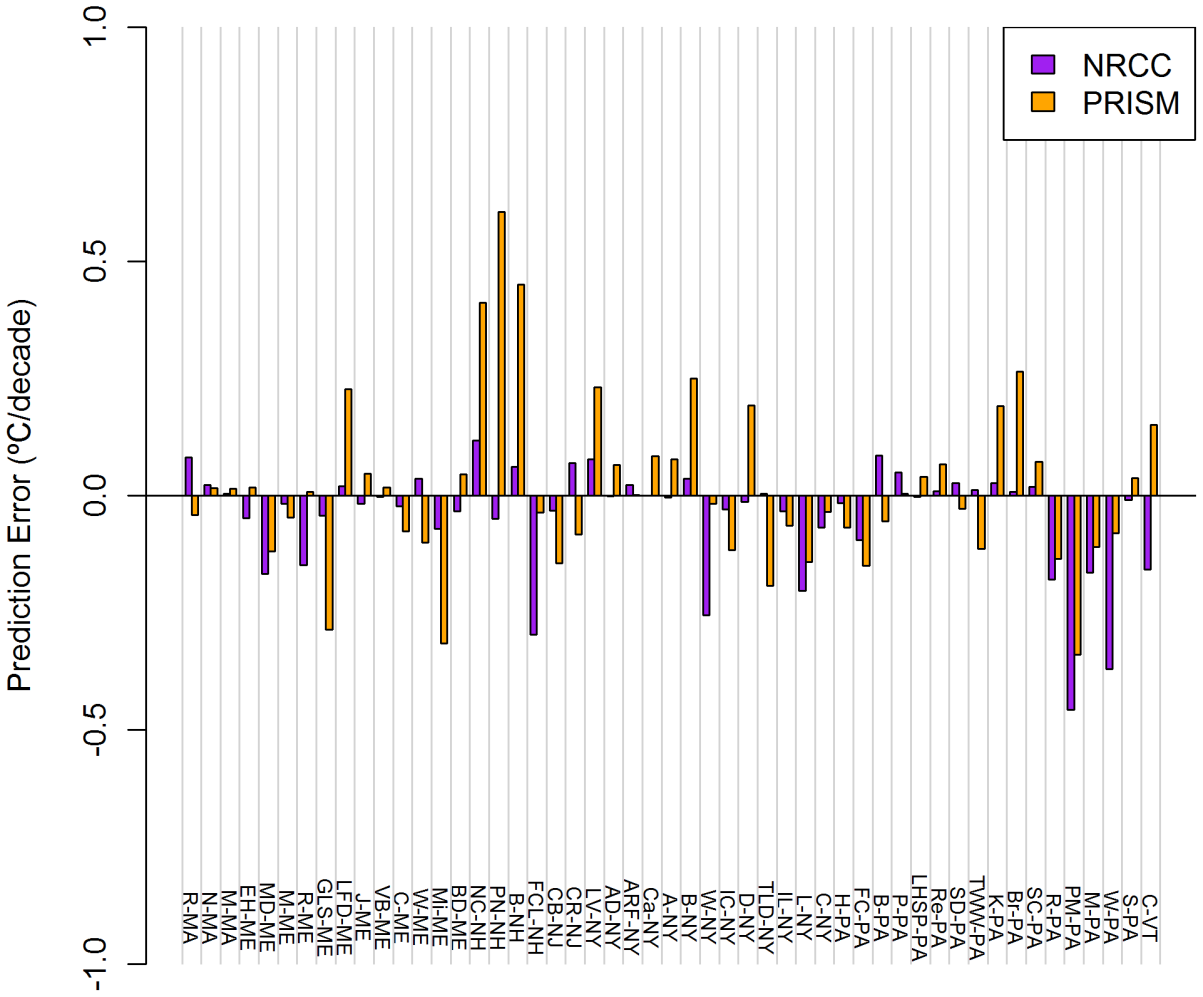
Mean TMax Trend prediction errors for NRCC and PRISM at 51 COOP weather stations. Stations across the US Northeast during 1980–2009. Data presented are averages of 12 monthly errors.

**Figure 5:**
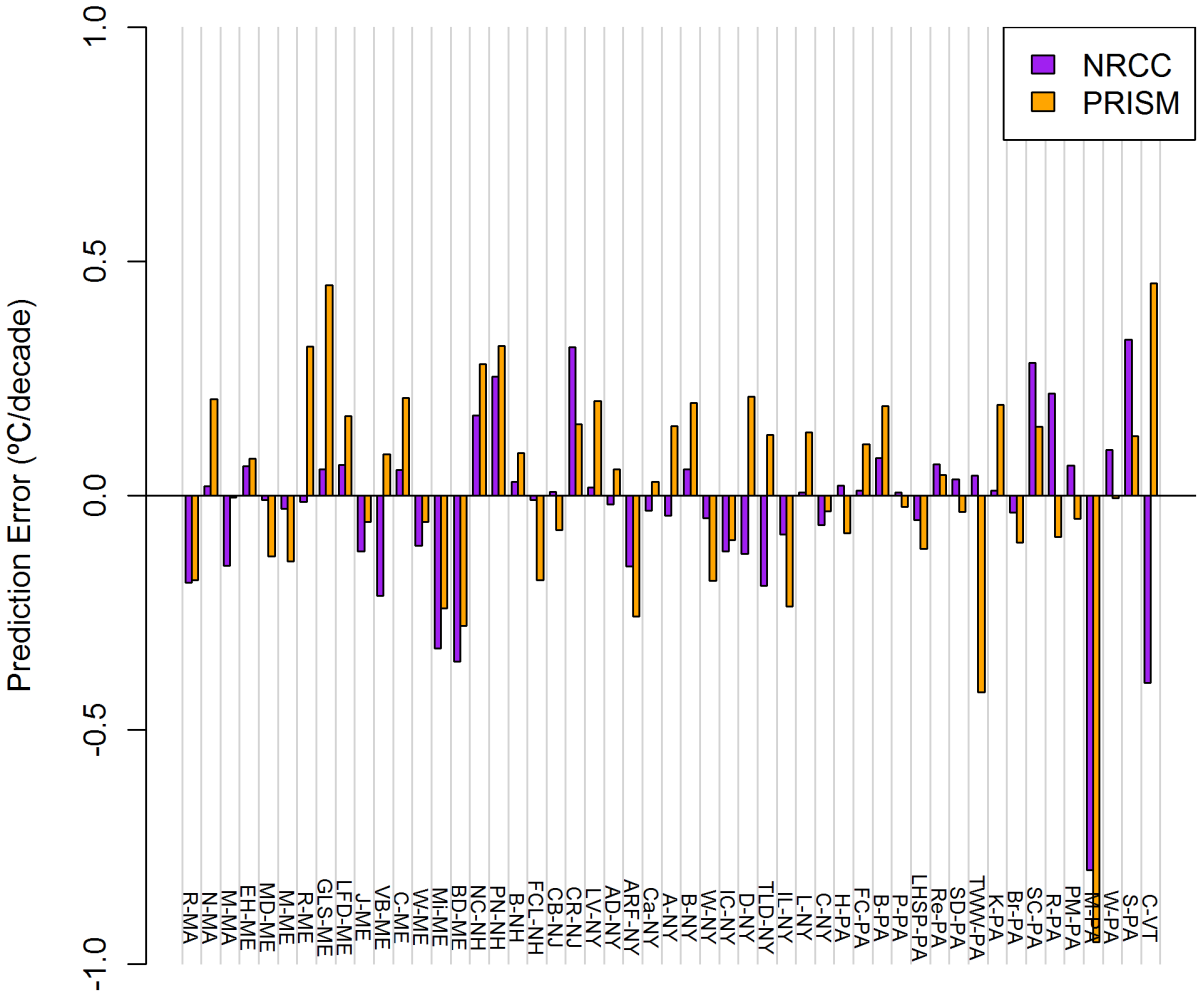
Mean TMin Trend prediction errors for NRCC and PRISM at 51 COOP weather stations. Stations across the US Northeast during 1980–2009. Data presented are averages of 12 monthly errors.

NRCC trend prediction errors ranged from −0.457 (PM-PA) to +0.118 (NC-NH) °C decade^−1^ for TMax (Fig. 4) and from −0.799 (M-PA) to +0.333 (S-PA) °C decade^−1^ for TMin (Fig. 5). Negative prediction errors for NRCC were largest for TMax during winter and for TMin during fall (Table 4).

PRISM trend prediction errors ranged from −0.339 (PM-PA) to 0.605 (PN-NH) °C decade^−1^ for TMax (Fig. 4) and from −0.952 (M-PA) to 0.453 (C-VT) °C decade^−1^ for TMin (Fig. 5). Positive prediction errors for PRISM were largest in summer for both TMax and TMin (Table 4).

### 3. Landscape Analysis of GHC Error

To evaluate effects of landscape factors on local-scale GHC prediction error, we conducted a general linear model comparison using all possible combinations of explanatory variables: latitude, elevation, distance to coast and interaction terms (Table 3).

Overall, linear models provided scant evidence for consistent spatial sources of uncertainty in the temperature means and trends predicted by either PRISM or NRCC. In several model comparisons, the null model had the most support (lowest AIC_c_), and in nearly every case the null was within 2.0 units AIC_c_ of the best model. Nearly all of the supported models had very low explanatory power (R^2^<0.2), indicating that these variables explained little of the observed variance in prediction errors at station locations across the region.

For predicting NRCC mean TMin error, models containing both *distance to coast* and *latitude* consistently were among the best for all seasons. In contrast, the PRISM mean TMin model containing only the *distance to coast* term performed among the best for all four seasons. For NRCC TMin trend error, a model with only *latitude* was best for all seasons, while for PRISM TMin trend error, the null model is consistently among the best for all four seasons (Tables S2.1–S2.8). As noted above, these models explained very little of the variability in PRISM and NRCC prediction error observed across the region.

**Figure 6:**
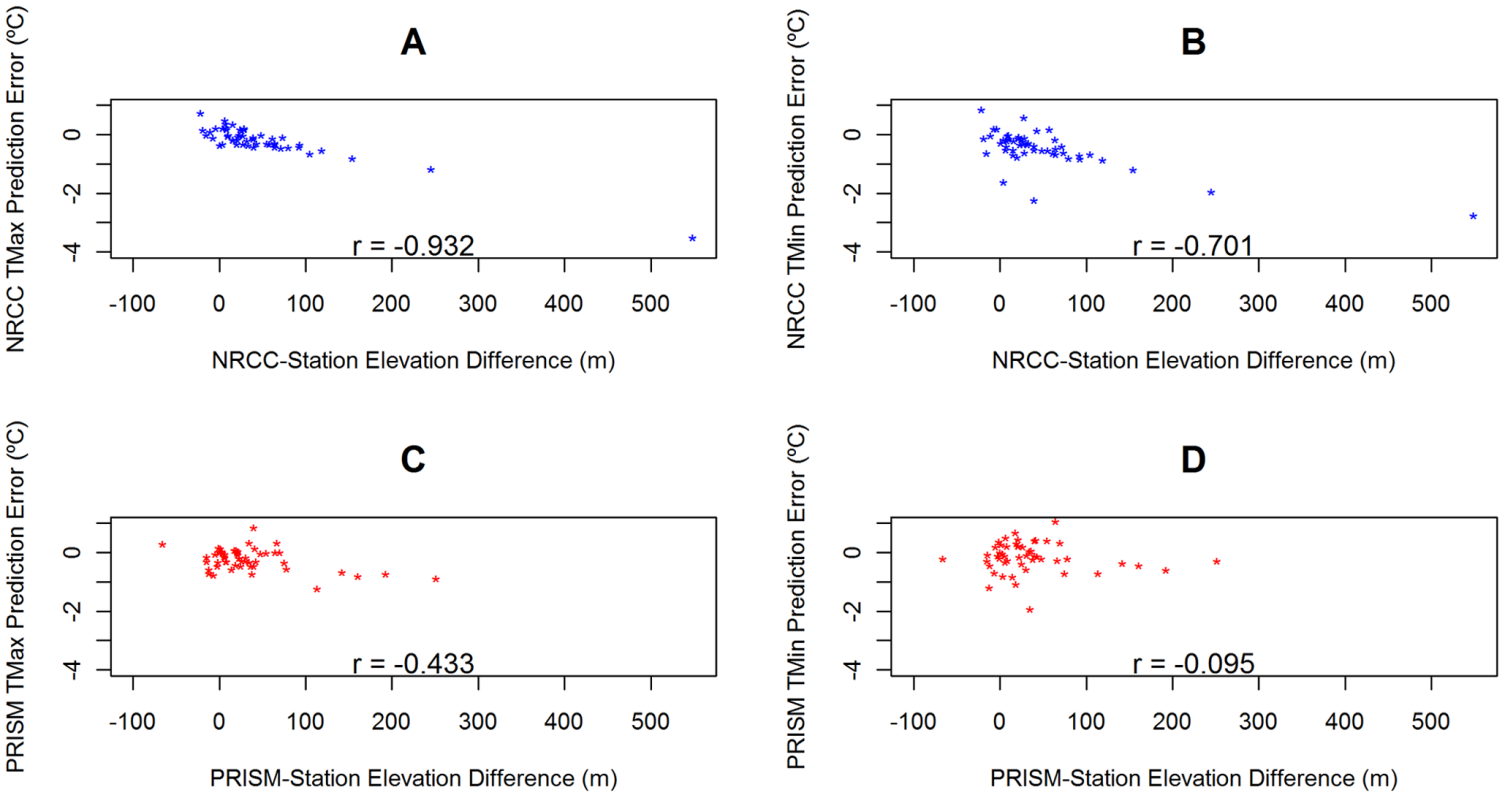
Relationships between GHC annual mean prediction error and DEM error. Plots for relationships between differences between DEM elevation estimates and station elevation (x-axis) and a) NRCC TMax Mean, b) NRCC TMin Mean, c) PRISM TMax Mean, and d) PRISM TMin Mean prediction errors. Plots shown all have Pearson’s correlation coefficients (r) labeled at the bottom of each plot.

Errors in digital elevation models were strongly negatively correlated with prediction errors for NRCC mean TMax (r = −0.93; p<0.0001) and mean TMin (r = −0.70; p<0.001), indicating that when NRCC increasingly overestimates elevation, its temperature predictions are increasingly colder than observed. For PRISM, TMax prediction error was also significantly correlated with its DEM errors, but to a lesser degree than NRCC (Fig. 6), while PRISM mean TMin error was not significantly correlated with DEM error. Neither of the trend estimate error sets for NRCC or PRISM were correlated with DEM error (all p>0.05).

### 4. Evaluating GHC Products at Local Scale

**Figure 7:**
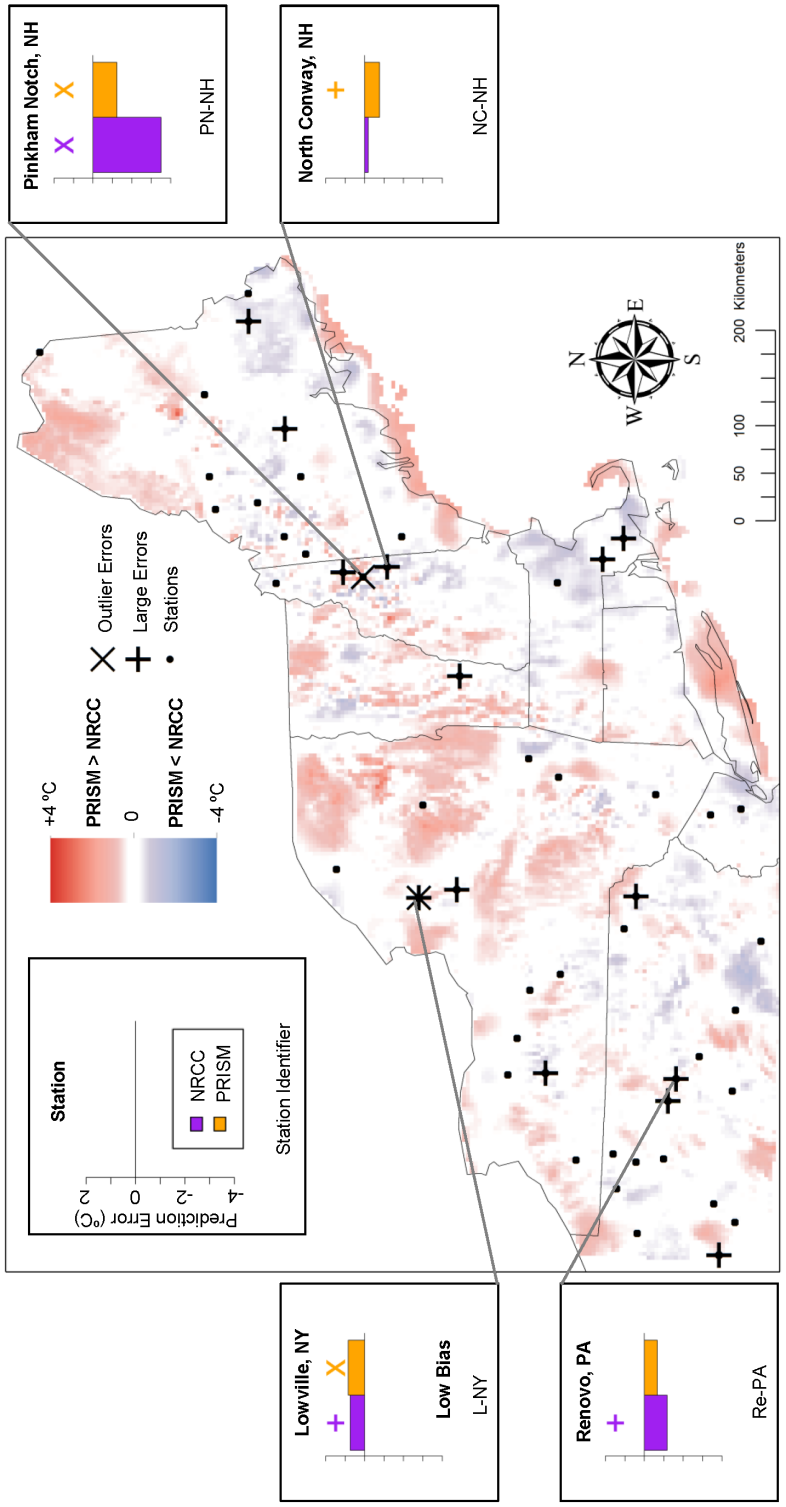
Mean TMax prediction error: Locations of large prediction errors. Error locations are in relation to areas of locally high and low temperature differences (PRISM-NRCC). Inset graphs display associated prediction errors for both GHC products, with a cross above large prediction errors (above ±1 SD) and an ‘X’ above prediction error outliers (above ±2 SD). Each station has two prediction errors plotted: purple (NRCC) and orange (PRISM).

**Figure 8:**
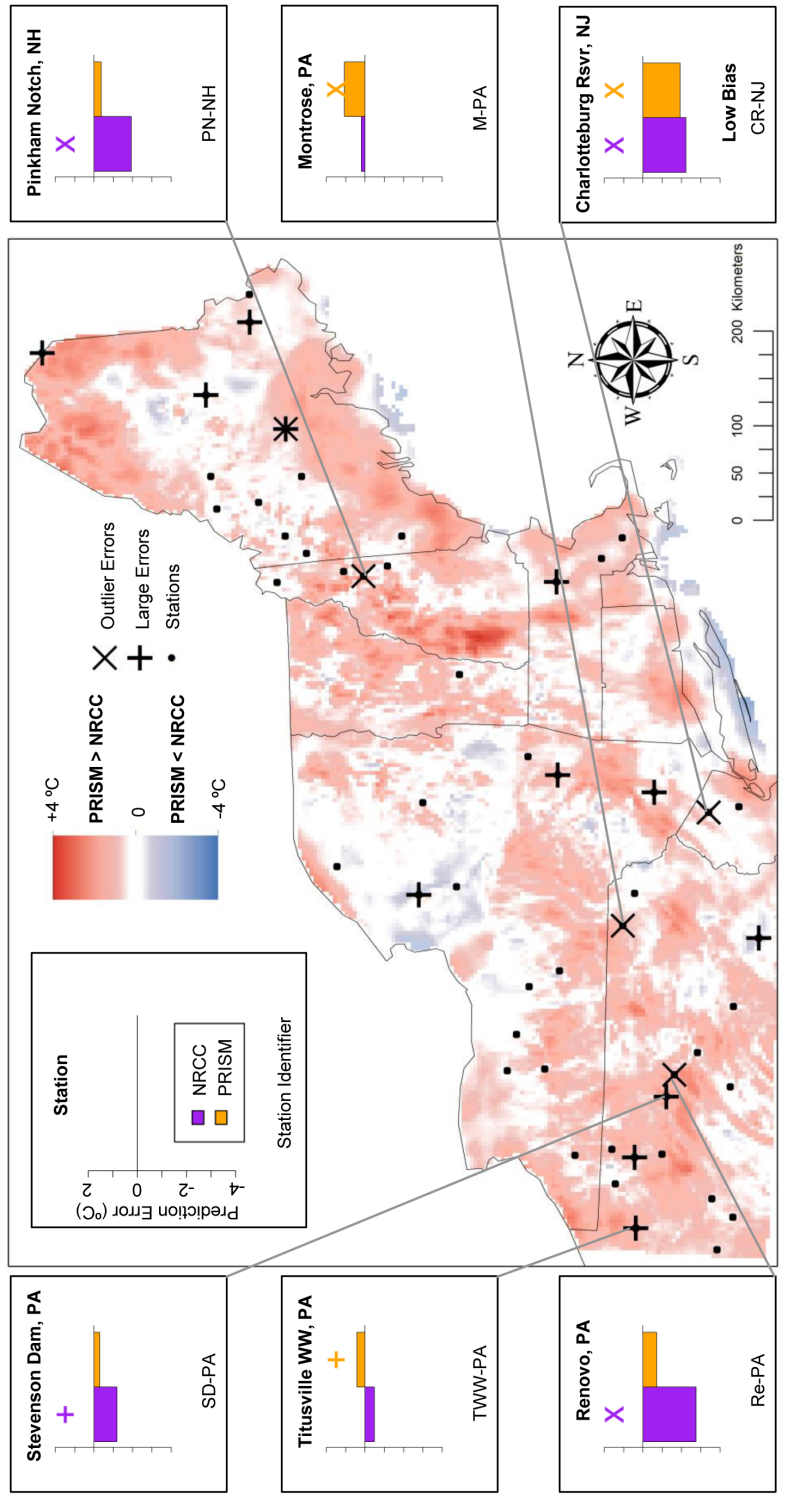
Mean TMin prediction error: Locations of large prediction errors. Error locations are in relation to areas of locally high and low temperature differences (PRISM-NRCC). Inset graphs display associated prediction errors for both GHC products, with a cross above large prediction errors (above ±1 SD) and an ‘X’ above prediction error outliers (above ±2 SD). Each station has two prediction errors plotted: purple (NRCC) and orange (PRISM).

**Figure 9:**
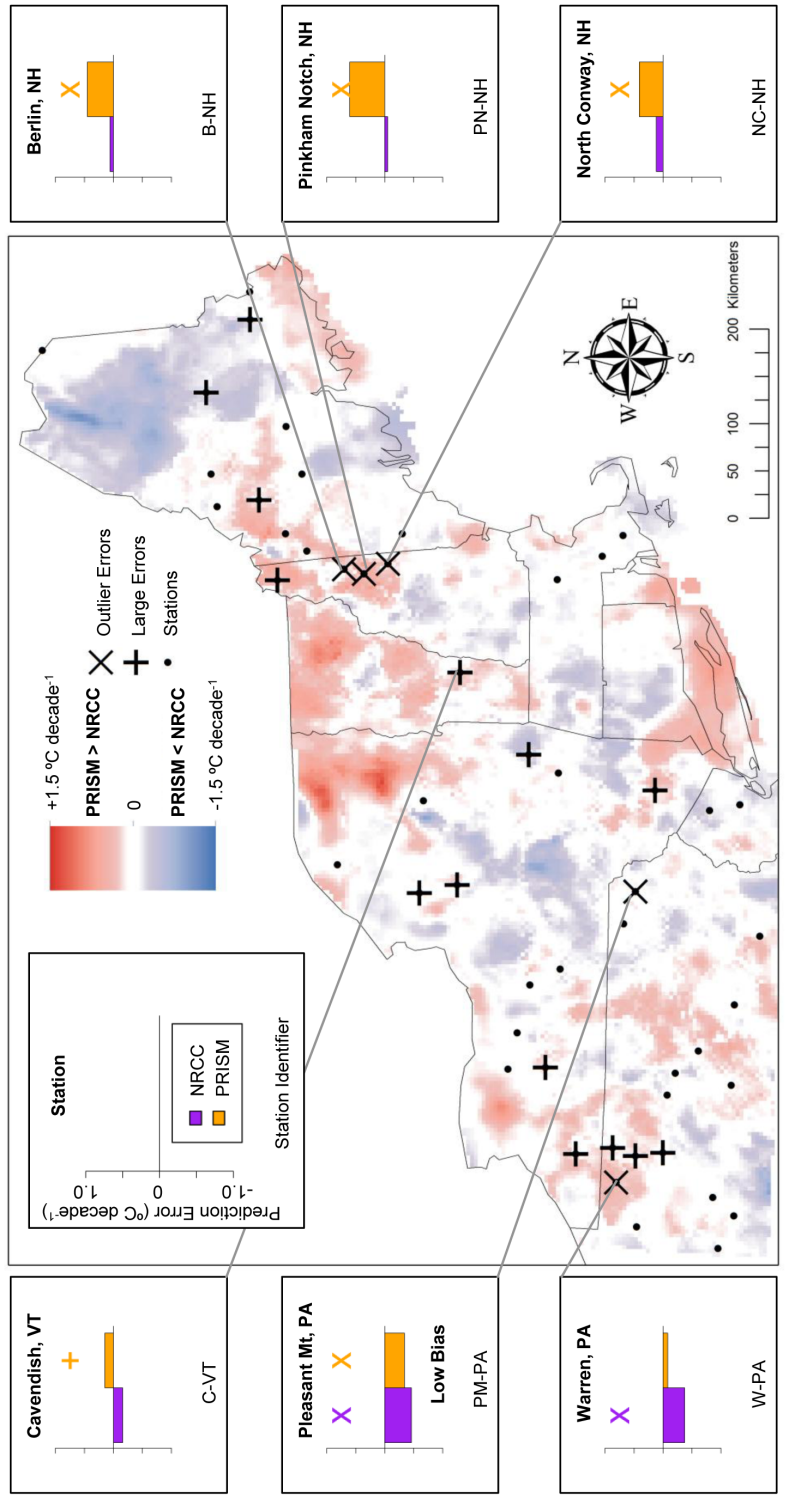
TMax Trend prediction error: Locations of large prediction errors. Error locations are in relation to areas of locally high and low temperature differences (PRISM-NRCC). Inset graphs display associated prediction errors for both GHC products, with a cross above large prediction errors (above ±1 SD) and an ‘X’ above prediction error outliers (above ±2 SD). Each station has two prediction errors plotted: purple (NRCC) and orange (PRISM).

**Figure 10:**
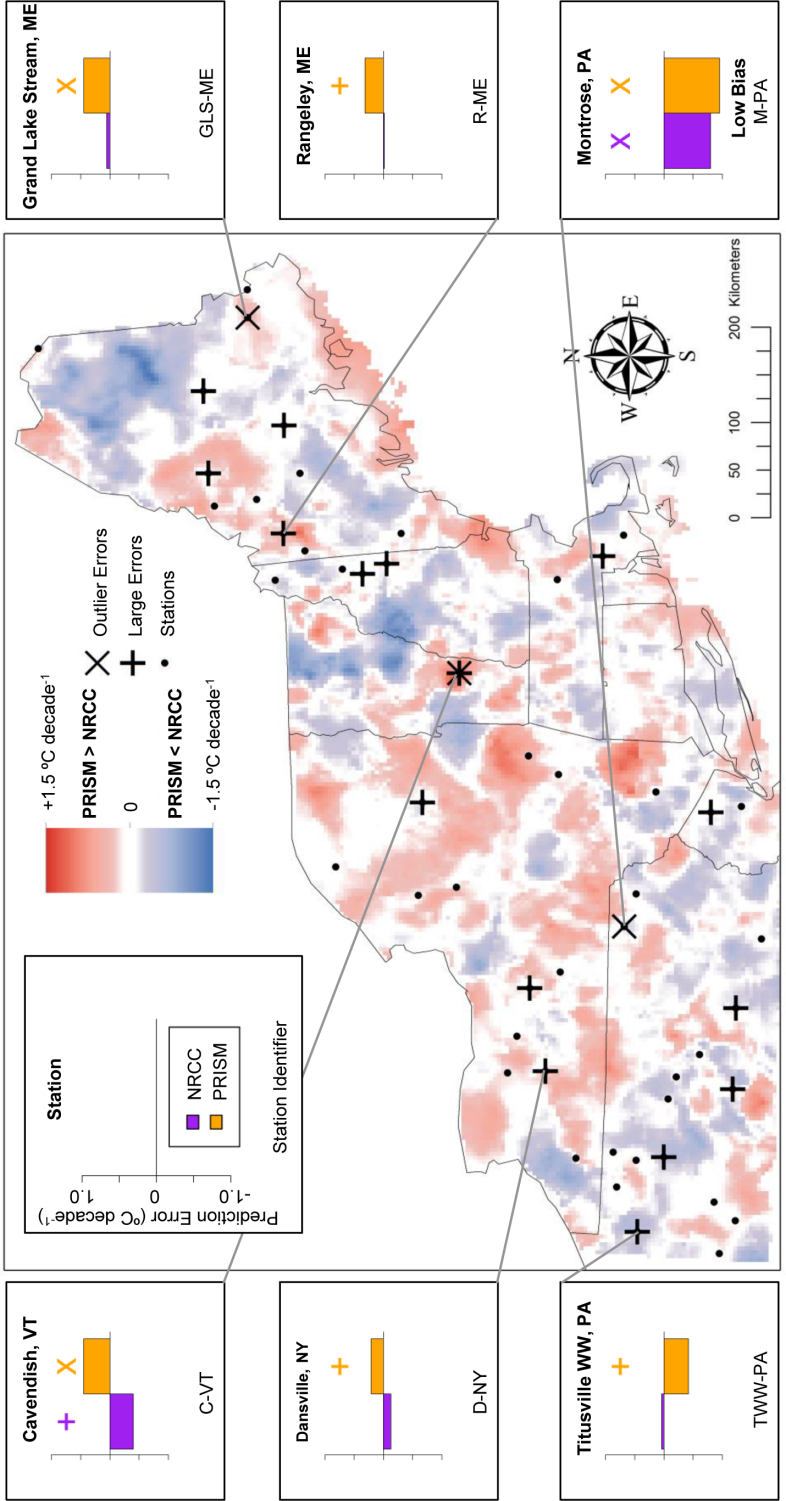
TMin Trend prediction error: Locations of large prediction errors. Error locations are in relation to areas of locally high and low temperature differences (PRISM-NRCC). Inset graphs display associated prediction errors for both GHC products, with a cross above large prediction errors (above ±1 SD) and an ‘X’ above prediction error outliers (above ±2 SD). Each station has two prediction errors plotted: purple (NRCC) and orange (PRISM).

We identified large outlier errors of NRCC and PRISM at weather stations and used these in conjunction with smaller errors as reference points to evaluate areas of disagreement between NRCC and PRISM. Using this approach, we found cases where the two products disagreed but one was clearly more representative of local conditions (i.e., mean temperature or trend magnitude during 1980–2009). There were also cases where PRISM and NRCC disagreed, but both had large errors and neither closely represented local conditions. For each set of analyses of TMax and TMin, these cases are depicted in Figures 7–10 and briefly described below.

For mean TMax, there was one station where large outlier errors coincided with a zone of disagreement in which PRISM>NRCC (Fig. 7). At Pinkham’s Notch, NH both PRISM and NRCC had large outlier errors but the NRCC error (−3.501°C) was much larger than the PRISM error (−1.227°C). Within a PRISM<NRCC zone, at the North Conway, NH station (NC-NH) PRISM had large negative error while the NRCC error was relatively small.

For mean TMin, the 5 stations identified with the largest PRISM-NRCC bias were all located in PRISM>NRCC zones (Fig. 8). In two of these cases NRCC had an outlier error that predicted much colder than observed. At Montrose, PA (M-PA), PRISM had a large positive outlier error while NRCC closely predicted the observed mean temperature. At Charlotteburg Reservoir (CR-NJ), both PRISM and NRCC had an outlier error, only slightly smaller than the NRCC error. This station was not located within a zone of disagreement, as the two products were in close agreement of each other.

For TMax trends, all of the 5 stations with the largest PRISM-NRCC bias magnitude were within PRISM>NRCC zones, i.e., where PRISM predicted more positive (warming) trends relative to NRCC (Fig. 9). PRISM had large positive outlier errors at three stations in close proximity in NH (B-NH, PN-NH, NC-NH). In northwestern PA, NRCC had a large negative outlier while PRISM closely predicted the observed trend (W-PA). However, in northeastern PA, both PRISM and NRCC were in relative agreement, exhibiting large negative outliers (PM-PA).

Lastly for TMin trends, 4 of the 5 high bias stations were in PRISM>NRCC zones (Fig. 10). At Cavendish, VT (C-VT), the two products predicted different trend directions but at similar magnitudes (rates). One station (TWW-PA) was within a PRISM<NRCC zone, as PRISM exhibited a large negative error and NRCC predicted relatively close to the observed trend. For the sixth station in Montrose, PA (M-PA), both products had large negative outliers, which was not within a zone of disagreement.

## Discussion

The increasing proliferation and use of high-resolution spatial climate data products has largely outpaced efforts to assess their uncertainty for various applications. Few guidelines exist for using these modeled data [14] and most of the GHC products are not provided to users with any estimates of uncertainty. We suggest that assessing this uncertainty, even at a basic level as we have done in this study, can be helpful for understanding strengths and limitations of these high-resolution gridded historical climate (GHC) products.

Our results based on 51 weather stations across the US Northeast suggest a relatively low degree of uncertainty in GHC predictions, but that this uncertainty varied in complex ways. On average, NRCC predicted lower than observed (cooler) for both means and trends, while PRISM predicted lower than observed for means and higher than observed for trends. Error varied by seasons and both PRISM and NRCC products had the largest errors during winter (DJF).

Although comparisons of overall prediction accuracy could be drawn from these results, our purpose was not to determine which product was ‘best’ for the US Northeast region. In fact, our results confirmed that, whether focusing on maximum or minimum temperatures, or means or trends, in different cases PRISM or NRCC might more accurately represent local conditions. In a few cases, both products had large errors that, depending on the application, may be considered unsatisfactory for representing local conditions. Mean error estimates were often biased by these large outliers and therefore median errors may better represent the overall uncertainty of a GHC product. However, these large outlier errors indicate that some degree of caution should be taken when applying these GHC data in research and decision-making.

Despite some prior indications of greater GHC uncertainty with increasing elevation and proximity to coast for the US Northeast [17], we found little evidence that these factors represented systematic sources of uncertainty in either PRISM or NRCC. Despite this result, there remains some circumstantial evidence that high elevation predictions, particularly those in rugged terrain, may be more erroneous. Our modeling was constrained by the lack of high elevation weather stations in the US Northeast, since we could not include any information at points above the highest elevation station located at 613 m (PN-NH).

However, elevation cannot be ruled out as a source of GHC uncertainty. We found that GHC errors may be a result of coarse-scale (e.g., 4 km) digital elevation models that inaccurately represent terrain [14], as well as modeling errors from interpolation or smoothing procedures [8]. For the US Northeast, we found that one product (NRCC) was more sensitive to these DEM errors than the other (PRISM). This result may be due to the different station weighting schemes used. PRISM employs a climate-elevation regression for each individual DEM grid cell, which assigns weights dependent on each weather station’s climatological similarity to the grid cell [3]. This methodology appears to reduce the sensitivity of PRISM TMax and TMin prediction errors to DEM error, relative to the NRCC product. NRCC uses modeled temperature lapse rates to interpolate gridded estimates to the weather station points, then bias-corrects the temperature fields based on station observations [8]. Our findings suggest the lapse rate calculations at elevations that are higher than actual elevation are likely the source of the error, since NRCC became increasingly colder than observed as its DEM error increased. We note that this sensitivity was apparent for mean temperature predictions but was absent for trend estimates, suggesting that DEM-related error does not explain uncertainty in GHC-based trend estimates. This is not surprising, as trend estimates would only be altered by temporal variability in interpolation methodology and each product’s DEM is constant throughout each time series.

As GHC users ourselves, we anticipate that ground-truthing analyses will support more informed decisions among the different GHC products available. While there are many considerations for choosing a GHC product, one of the primary concerns will likely be whether the local predictions are more or less consistent with local observations. Our approach of mapping large outlier errors with spatial zones of cross-product disagreement (PRISM-NRCC bias) provides a basis to make decisions about which GHC product might be more reliable in a local context. Results of this study suggest that such decisions may vary depending on whether the variables of interest represent average conditions or metrics of change. For example, in the case of mean temperatures, in zones where PRISM was consistently predicting higher temperatures than NRCC, we observed that it was often because NRCC had large negative errors (i.e., predicted much cooler than the station). For temperature trends, by contrast, PRISM typically had large positive errors (i.e., predicted warmer trends) while NRCC predictions were often closer to station observations, with a few exceptions.

At Pinkham’s Notch, NH (PN-NH), where PRISM and NRCC differed significantly for both means and trends, we found that PRISM more accurately predicted the mean TMax (Fig. 7) while NRCC more accurately predicted the TMax trend, despite both products exhibiting a large outlier error (Fig. 9). Therefore, if GHC users are interested in assessing both temperature means and trends with highest local-scale accuracy, it may be appropriate to use both PRISM and NRCC, respectively. Despite the potential complexities that such an approach poses, our results suggest that incorporating several spatial climate datasets into a project – which is akin to comparing multiple alternative models [20] – can enhance the use and interpretation of these products, especially at local scales.

Lastly, although we did not question the accuracy of reference data from COOP weather stations, these observations may be affected by instrumental changes and environmental (site) modifications [30]. Instrumentation changes or alteration of measurement schedules can result in biases of>±1°C at individual stations [31], while observation-time adjustments may be used robustly in some cases [32]. NRCC accounts for this potential bias, using stations with a specific observation times within their daily 8 am to 8 am Local Standard Time (LST) observation window [8]. PRISM uses stations with a variety of time steps ranging from hourly to yearly, with the daily station data observation times varying over a range of time steps [3]. Maximum and minimum temperature estimates, as well as precipitation totals, could vary on a daily basis in the source data for these two products. An analysis evaluating the impacts of the source data would produce very applicable uncertainty estimates; however a specific spatial and temporal record of PRISM source data is not readily available for public users. These records could be used to identify data to download from the source’s official platforms.

Other sources of error in weather station records include: warm bias in nighttime minimum temperatures, poor site location, failure to account for the effects of wind and humidity on temperature trends, uncertainties in homogenization of surface temperature data, and the influences of land use and land cover change on trends; most of these are poorly understood [33]. Despite these possible issues, we treated COOP station data as “truth” because their role as source data in the modeling of both NRCC and PRISM designated them as an ideal reference point. Other local reference points of value may include independent weather record (outside of the COOP network) as well as long-term phenological observations such as lake and river ice, migratory arrivals, and the emergence of insects or spring foliage.

### Conclusions

Our study points to recommendations for both GHC users and GHC producers. First, GHC products may incorporate the same source data differently, creating output that can disagree considerably with both the source and other products. We suggest that GHC users become more familiar with the underlying models and prediction capabilities before utilizing these products, especially in cases where the GHC data takes the place of an independent (explanatory) variable in statistical or simulation models. This should include a stronger grasp of the fundamental tradeoff between resolution and realism in spatial climate data, a concept that is well-documented for GHC datasets [14].

Second, although there is some evidence that GHC uncertainty may be related to elevation or geographic factors [17], we found no consistent or significant effects of these factors on GHC prediction error across the US Northeast. However, we found evidence that climate-elevation interpolation techniques, namely the selection of a DEM, have a strong impact on GHC prediction error for mean temperatures. While estimating these errors is straightforward, GHC users cannot evaluate uncertainty for the vast majority of raster cells without a corresponding climate record, which does not exist [2].

Third, we suggest that GHC producers could provide metadata with their products, such as the inclusion of confidence intervals [2] or bias fields [8] that are generated by their modeling process, as they would be of great value to GHC users applying these data in research and decision-making. Numerous techniques exist that allow researchers to incorporate these uncertainty estimates in statistical, spatial and simulation models, allowing the necessary propagation of error in their model frameworks [20]. In decision-making situations, these uncertainty estimates can provide bounds for scenario development and/or evaluation, and provide a richer basis for users to decide among GHC products for local applications.

Lastly, we encourage GHC users weigh the available uncertainty estimates and related information associated with GHC products and to consider using multiple alternative climate datasets, model outputs and information sources in any scientific application of GHC products. Whenever possible, utilizing a non-modeled (measured) reference data source, as displayed in this study, is helpful for deciding which GHC product may be best utilized for a given location and purpose.

## Supporting Information

Table S1 List of stations with associated acronyms used within the text of the paper, Station IDs, geographic coordinates (longitude, latitude), and raw landscape factors (elevation, coastal distance) for each instrumental data source.
(PDF)

Table S2 **Table S2.1.** NRCC Mean Tmax prediction error model(s) within 2 corrected Akaike Information Criterion (AICc) units of lowest AIC among 13 candidate general linear models over US Northeast, sorted by dAICc, with Akaike weights and coefficient of determination (R2), for a) Winter, b) Spring, c) Summer, d) Fall. **Table S2.2.** NRCC Mean Tmin prediction error model(s) within 2 corrected Akaike Information Criterion (AICc) units of lowest AIC among 13 candidate general linear models over US Northeast, sorted by dAICc, with Akaike weights and coefficient of determination (R2), for a) Winter, b) Spring, c) Summer, d) Fall. Table S2.3. PRISM Mean Tmax prediction error model(s) within 2 corrected Akaike Information Criterion (AICc) units of lowest AIC among 13 candidate general linear models over US Northeast, sorted by dAICc, with Akaike weights and coefficient of determination (R2), for a) Winter, b) Spring, c) Summer, d) Fall. **Table S2.4.** PRISM Mean Tmin prediction error model(s) within 2 corrected Akaike Information Criterion (AICc) units of lowest AIC among 13 candidate general linear models over US Northeast, sorted by dAICc, with Akaike weights and coefficient of determination (R2), for a) Winter, b) Spring, c) Summer, d) Fall. **Table S2.5.** NRCC Tmax Trend prediction error model(s) within 2 corrected Akaike Information Criterion (AICc) units of lowest AIC among 13 candidate general linear models over US Northeast, sorted by dAICc, with Akaike weights and coefficient of determination (R2), for a) Winter, b) Spring, c) Summer, d) Fall. **Table S2.6.** NRCC Tmin Trend prediction error model(s) within 2 corrected Akaike Information Criterion (AICc) units of lowest AIC among 13 candidate general linear models over US Northeast, sorted by dAICc, with Akaike weights and coefficient of determination (R2), for a) Winter, b) Spring, c) Summer, d) Fall. **Table S2.7.** PRISM Tmax Trend prediction error model(s) within 2 corrected Akaike Information Criterion (AICc) units of lowest AIC among 13 candidate general linear models over US Northeast, sorted by dAICc, with Akaike weights and coefficient of determination (R2), for a) Winter, b) Spring, c) Summer, d) Fall. **Table S2.8.** PRISM Tmin Trend prediction error model(s) within 2 corrected Akaike Information Criterion (AICc) units of lowest AIC among 13 candidate general linear models over US Northeast, sorted by dAICc, with Akaike weights and coefficient of determination (R2), for a) Winter, b) Spring, c) Summer, d) Fall.
(PDF)
