# Supplementary material for: Assessing Uncertainty in High-Resolution Spatial Climate Data across the US Northeast
Source: PLoS One. 2013 Aug 1;8(8):e70260. doi: 10.1371/journal.pone.0070260 (PMC3731317; doi:10.1371/journal.pone.0070260)
Supplement: Table S1 — List of stations with associated acronyms used within the text of the paper, Station IDs, geographic coordinates (longitude, latitude), and raw landscape factors (elevation, coastal distance) for each instrumental data source. (PDF) [file pone.0070260.s001.pdf]

Station Name	*Acronym*	*Station ID*	*Longitude*	*Latitude*	*Elevation (m)*	*Coastal Distance (m)*
Rochester, MA	R-MA	196938	-70.918	41.785	19.8	20377.3
Norton, MA	N-MA	195984	-71.167	41.993	29	27870.3
Maynard, MA	M-MA	194580	-71.442	42.429	62.5	41850.2
Corinna, ME	C-ME	171628	-69.242	44.92	90.5	57896.8
Woodland, ME	W-ME	179891	-67.404	45.157	42.7	29510.4
Millinocket, ME	Mi-ME	175304	-68.705	45.65	109.7	123193.7
Brassua Dam, ME	BD-ME	170814	-69.812	45.66	323.1	148289.6
East Hiram, ME	EH-ME	172238	-70.754	43.879	160.9	52358
Middle Dam, ME	MD-ME	175261	-70.917	44.793	445	130123.1
Madison, ME	M-ME	174927	-69.888	44.798	79.2	88515.7
Rangeley, ME	R-ME	177037	-70.672	44.989	466.3	137449.2
Grand Lake Stream, ME	GLS-ME	173261	-67.774	45.178	88.4	58922.6
Long Falls Dam, ME	LFD-ME	171870	-70.198	45.222	353.6	132888.1
Jackman, ME	J-ME	174086	-70.255	45.623	362.7	166538.7
Van Buren, ME	VB-ME	178965	-67.94	47.166	139	226558.4
First Connecticut Lake, NH	FCL-NH	272999	-71.287	45.087	506	171519.1
North Conway, NH	NC-NH	275995	-71.138	44.03	165.8	85295.3
Pinkham Notch, NH	PN-NH	276818	-71.255	44.263	612.6	107147
Berlin, NH	B-NH	270690	-71.184	44.449	283.5	113247.5
Charlotteburg Rsvr, NJ	CR-NJ	281582	-74.423	41.035	231.6	44049
Canoe Brook, NJ	CB-NJ	281335	-74.354	40.744	54.9	19626.6
Walden, NY	W-NY	308906	-74.163	41.551	115.8	76562
Ithaca Cornell, NY	IC-NY	304174	-76.449	42.449	292.6	271005.8
Dansville, NY	D-NY	301974	-77.718	42.566	201.2	363900.3
Troy L&D, NY	TLD-NY	308600	-73.683	42.75	7.3	172141.4
Indian Lake, NY	IL-NY	304102	-74.269	43.755	506	296603.7
Lowville, NY	L-NY	304912	-75.482	43.797	262.1	344518.8
Canton, NY	C-NY	301185	-75.11	44.577	136.6	393935.4
Little Valley, NY	LV-NY	304808	-78.812	42.247	495.3	424676
Alcove Dam, NY	AD-NY	300063	-73.927	42.47	185	156572.7

Aurora Rsch Farm, NY	ARF-NY	300331	-76.659	42.734	253	304827.3
Canandaigua, NY	Ca-NY	301152	-77.281	42.845	219.5	352837.4
Avon, NY	A-NY	300343	-77.756	42.92	166.1	390725
Boonville, NY	B-NY	300785	-75.37	43.436	472.4	303537.1
State College, PA	SC-PA	368449	-77.867	40.793	356.6	307362.4
Ridgway, PA	R-PA	367477	-78.75	41.42	414.5	393937.1
Pleasant Mt, PA	PM-PA	367029	-75.446	41.739	548.6	157578.5
Montrose, PA	M-PA	365915	-75.858	41.851	432.8	193204.4
Warren, PA	W-PA	369298	-79.15	41.85	368.8	436194.4
Selinsgrove, PA	S-PA	367931	-76.861	40.783	128	223297.3
Hamburg, PA	H-PA	363632	-75.995	40.552	116.7	148427.8
Ford City, PA	FC-PA	362942	-79.5	40.717	283.5	446815.4
Butler, PA	B-PA	361139	-79.917	40.85	304.8	482195.6
Putneyville, PA	P-PA	367229	-79.282	40.925	390.1	426908.1
Lock Haven Sewege Plant, PA	LHSP-PA	365109	-77.45	41.117	172.5	277381.8
Renovo, PA	Re-PA	367409	-77.738	41.33	201.2	306457.9
Stevenson Dam, PA	SD-PA	368469	-78.017	41.4	284.1	332315.8
Titusville Wtr Works, PA	TWW-PA	368888	-79.7	41.633	371.9	473306.2
Kane, PA	K-PA	364432	-78.804	41.677	533.4	404431.7
Bradford, PA	Br-PA	360868	-78.714	41.898	506	404869
Cavendish, VT	C-VT	431243	-72.599	43.385	256.6	161322.1
