# Supplementary material for: Assessing Uncertainty in High-Resolution Spatial Climate Data across the US Northeast
Source: PLoS One. 2013 Aug 1;8(8):e70260. doi: 10.1371/journal.pone.0070260 (PMC3731317; doi:10.1371/journal.pone.0070260)
Supplement: Table S2 — 1. NRCC Mean Tmax prediction error model(s) within 2 corrected Akaike Information Criterion (AICc) units of lowest AIC among 13 candidate general linear models over US Northeast, sorted by dAICc, with Akaike weights and coefficient of determination (R2), for a) Winter, b) Spring, c) Summer, d) Fall. Table S2.2. NRCC Mean Tmin prediction error model(s) within 2 corrected Akaike Information Criterion (AICc) units of lowest AIC among 13 candidate general linear models over US Northeast, sorted by dAICc, with Akaike weights and coefficient of determination (R2), for a) Winter, b) Spring, c) Summer, d) Fall. Table S2.3. PRISM Mean Tmax prediction error model(s) within 2 corrected Akaike Information Criterion (AICc) units of lowest AIC among 13 candidate general linear models over US Northeast, sorted by dAICc, with Akaike weights and coefficient of determination (R2), for a) Winter, b) Spring, c) Summer, d) Fall. Table S2.4. PRISM Mean Tmin prediction error model(s) within 2 corrected Akaike Information Criterion (AICc) units of lowest AIC among 13 candidate general linear models over US Northeast, sorted by dAICc, with Akaike weights and coefficient of determination (R2), for a) Winter, b) Spring, c) Summer, d) Fall. Table S2.5. NRCC Tmax Trend prediction error model(s) within 2 corrected Akaike Information Criterion (AICc) units of lowest AIC among 13 candidate general linear models over US Northeast, sorted by dAICc, with Akaike weights and coefficient of determination (R2), for a) Winter, b) Spring, c) Summer, d) Fall. Table S2.6. NRCC Tmin Trend prediction error model(s) within 2 corrected Akaike Information Criterion (AICc) units of lowest AIC among 13 candidate general linear models over US Northeast, sorted by dAICc, with Akaike weights and coefficient of determination (R2), for a) Winter, b) Spring, c) Summer, d) Fall. Table S2.7. PRISM Tmax Trend prediction error model(s) within 2 corrected Akaike Information Criterion (AICc) units of lowest AIC among [file pone.0070260.s002.pdf]

Table S2.1. NRCC Mean Tmax prediction error model(s) within 2 corrected Akaike Information Criterion (AICc) units of lowest AIC among 13 candidate general linear models over US Northeast, sorted by dAICc, with Akaike weights and coefficient of determination (R2), for a) Winter, b) Spring, c) Summer, d) Fall.

a) Winter (DJA)

Model	ΔAICc	Weight	R2
Lat+Coast*Elev	0.0	0.74	0.98

b) Spring (MAM)

Model	ΔAICc	Weight	R2
Null	0.0	0.26	0.00
Coast	1.1	0.15	0.02
Elev+Coast	1.7	0.11	0.05
Elev	1.8	0.11	0.01

c) Summer (JJA)

Model	ΔAICc	Weight	R2
Coast*Elev	0.0	0.57	0.37
Lat+Coast*Elev	1.2	0.30	0.39

d) Fall (SON)

Model	ΔAICc	Weight	R2
Coast*Elev	0.0	0.58	0.41
Lat+Coast*Elev	1.9	0.23	0.42

Table S2.2. NRCC Mean Tmin prediction error model(s) within 2 corrected Akaike Information Criterion (AICc) units of lowest AIC among 13 candidate general linear models over US Northeast, sorted by dAICc, with Akaike weights and coefficient of determination (R2), for a) Winter, b) Spring, c) Summer, d) Fall.

a) Winter (DJA)

Model	ΔAICc	Weight	R2
Null	0.0	0.25	0.00
Coast	0.4	0.21	0.03
Lat+Coast	1.6	0.12	0.06
Lat	2.0	0.09	< 0.01

b) Spring (MAM)

Model	ΔAICc	Weight	R2
Lat+Elev+Coast	0.0	0.29	0.15
Lat	1.1	0.13	0.05
Null	1.5	0.10	0.00
Lat+Coast	1.6	0.10	0.09
Lat+Coast*Elev	1.6	0.10	0.17

c) Summer (JJA)

Model	ΔAICc	Weight	R2
Lat+Elev+Coast	0.0	0.23	0.17
Elev+Coast	1.1	0.13	0.11
Lat+Coast*Elev	1.4	0.11	0.19
Elev	1.7	0.10	0.05
Coast+Elev*Lat	1.9	0.09	0.18

d) Fall (SON)

Model	ΔAICc	Weight	R2
Lat+Elev+Coast	0.0	0.20	0.16
Lat+Coast	0.4	0.16	0.11
Lat	0.8	0.14	0.06
Lat+Coast*Elev	1.0	0.12	0.18
Null	1.7	0.09	0.00

Table S2.3. PRISM Mean Tmax prediction error model(s) within 2 corrected Akaike Information Criterion (AICc) units of lowest AIC among 13 candidate general linear models over US Northeast, sorted by dAICc, with Akaike weights and coefficient of determination (R2), for a) Winter, b) Spring, c) Summer, d) Fall.

a) Winter (DJA)

Model	ΔAICc	Weight	R2
Null	0.0	0.34	0.00
Coast	1.7	0.14	0.01

b) Spring (MAM)

Model	ΔAICc	Weight	R2
Null	0.0	0.20	0.00
Coast*Elev	0.5	0.16	0.12
Coast	0.9	0.13	0.03
Lat+Coast*Elev	1.9	0.08	0.14
Elev+Coast	1.9	0.08	0.05
Elev	2.0	0.08	0.01

c) Summer (JJA)

Model	ΔAICc	Weight	R2
Null	0.0	0.24	0.00
Coast	0.8	0.16	0.03
Elev*Lat	0.9	0.15	0.11

d) Fall (SON)

Model	ΔAICc	Weight	R2
Null	0.0	0.29	0.00
Elev	1.7	0.12	0.01
Elev*Lat	1.9	0.11	0.10

Table S2.4. PRISM Mean Tmin prediction error model(s) within 2 corrected Akaike Information Criterion (AICc) units of lowest AIC among 13 candidate general linear models over US Northeast, sorted by dAICc, with Akaike weights and coefficient of determination (R2), for a) Winter, b) Spring, c) Summer, d) Fall.

a) Winter (DJA)

Model	ΔAICc	Weight	R2
Null	0.0	0.35	0.00
Coast	1.7	0.15	0.01

b) Spring (MAM)

Model	ΔAICc	Weight	R2
Null	0.0	0.21	0.00
Lat	0.7	0.14	0.03
Lat+Coast	0.8	0.14	0.07
Coast	1.4	0.10	0.02

c) Summer (JJA)

Model	ΔAICc	Weight	R2
Null	0.0	0.25	0.00
Coast	1.1	0.15	0.02
Elev*Lat	1.7	0.11	0.10

d) Fall (SON)

Model	ΔAICc	Weight	R2
Null	0.0	0.28	0.00
Elev	1.8	0.11	0.01
Coast	1.9	0.11	0.01

Table S2.5. NRCC Tmax Trend prediction error model(s) within 2 corrected Akaike Information Criterion (AICc) units of lowest AIC among 13 candidate general linear models over US Northeast, sorted by dAICc, with Akaike weights and coefficient of determination (R2), for a) Winter, b) Spring, c) Summer, d) Fall.

a) Winter (DJA)

Model	ΔAICc	Weight	R2
Null	0.0	0.22	0.00
Elev+Coast	0.8	0.15	0.07
Elev	1.1	0.12	0.02
Coast	1.3	0.12	0.01
Lat	1.7	0.10	0.01
Coast*Elev	2.0	0.08	0.09

b) Spring (MAM)

Model	ΔAICc	Weight	R2
Coast*Elev	0.0	0.43	0.21

c) Summer (JJA)

Model	ΔAICc	Weight	R2
Elev	0.0	0.40	0.13
Lat+Elev	2.0	0.15	0.13

d) Fall (SON)

Model	ΔAICc	Weight	R2
Elev	0.0	0.31	0.10
Elev+Coast	1.1	0.18	0.12

Table S2.6. NRCC Tmin Trend prediction error model(s) within 2 corrected Akaike Information Criterion (AICc) units of lowest AIC among 13 candidate general linear models over US Northeast, sorted by dAICc, with Akaike weights and coefficient of determination (R2), for a) Winter, b) Spring, c) Summer, d) Fall.

a) Winter (DJA)

Model	ΔAICc	Weight	R2
Lat	0.0	0.28	0.06
Null	1.1	0.16	0.00
Lat+Elev	1.5	0.13	0.08

b) Spring (MAM)

Model	ΔAICc	Weight	R2
Lat	0.0	0.41	0.10
Lat+Elev	2.0	0.15	0.11

c) Summer (JJA)

Model	ΔAICc	Weight	R2
Null	0.0	0.23	0.00
Lat	0.2	0.21	0.04
Elev	1.4	0.11	0.02
Lat+Elev	1.7	0.10	0.06
Lat+Coast	2.0	0.08	0.05

d) Fall (SON)

Model	ΔAICc	Weight	R2
Null	0.0	0.32	0.00
Lat	0.8	0.22	0.03
Coast	2.0	0.12	0.01

Table S2.7. PRISM Tmax Trend prediction error model(s) within 2 corrected Akaike Information Criterion (AICc) units of lowest AIC among 13 candidate general linear models over US Northeast, sorted by dAICc, with Akaike weights and coefficient of determination (R2), for a) Winter, b) Spring, c) Summer, d) Fall.

a) Winter (DJA)

Model	ΔAICc	Weight	R2
Elev	0.0	0.26	0.06
Null	1.0	0.15	0.00
Coast	1.4	0.13	0.04
Elev+Coast	1.9	0.10	0.07

b) Spring (MAM)

Model	ΔAICc	Weight	R2
Elev	0.0	0.28	0.09
Elev*Lat	1.0	0.17	0.15
Elev+Coast	1.2	0.15	0.11

c) Summer (JJA)

Model	ΔAICc	Weight	R2
Elev*Lat	0.0	0.29	0.18
Elev	1.2	0.16	0.07
Lat+Elev	1.8	0.12	0.11

d) Fall (SON)

Model	ΔAICc	Weight	R2
Elev	0.0	0.27	0.06
Null	1.1	0.15	0.00
Elev+Coast	1.7	0.18	0.08
Elev*Lat	1.8	0.11	0.12
Lat+Elev	2.0	0.10	0.07

Table S2.8. PRISM Tmin Trend prediction error model(s) within 2 corrected Akaike Information Criterion (AICc) units of lowest AIC among 13 candidate general linear models over US Northeast, sorted by dAICc, with Akaike weights and coefficient of determination (R2), for a) Winter, b) Spring, c) Summer, d) Fall.

a) Winter (DJA)

Model	ΔAICc	Weight	R2
Null	0.0	0.37	0.00
Elev	1.7	0.16	0.01
Lat	2.0	0.14	0.01

b) Spring (MAM)

Model	ΔAICc	Weight	R2
Null	0.0	0.36	0.00
Elev	1.7	0.15	0.01
Coast	2.0	0.13	0.01

c) Summer (JJA)

Model	ΔAICc	Weight	R2
Null	0.0	0.38	0.00
Lat	1.8	0.16	0.01
Coast	2.0	0.14	< 0.01

d) Fall (SON)

Model	ΔAICc	Weight	R2
Null	0.0	0.31	0.00
Lat	1.1	0.18	0.02
Elev	1.6	0.14	0.01
